# Assessment of community perceptions and risk to common zoonotic diseases among communities living at the human-livestock-wildlife interface in Nakuru West, Kenya: A participatory epidemiology approach

**DOI:** 10.1371/journal.pntd.0011086

**Published:** 2023-01-26

**Authors:** Maurice Omondi Owiny, Ben Kipchumba Ngare, Bernard Chege Mugo, Jacob Rotich, Arithi Mutembei, Khadijah Chepkorir, Rinah Sitawa, Mark Obonyo, Joshua Orungo Onono

**Affiliations:** 1 Kenya Field Epidemiology and Laboratory Training Program, Ministry of Health, Nairobi, Kenya; 2 Department of Health, County Government of Nakuru, Nakuru, Kenya; 3 Department of Agriculture, Wajir County Government, Wajir, Kenya; 4 Regional Veterinary Laboratory, Uasin Gishu County, Eldoret, Kenya; 5 Ministry of Agriculture, Livestock and Fisheries, Nairobi, Kenya; 6 Food and Agriculture Organization of the United Nations, Nairobi, Kenya; 7 Department of Public Health, Pharmacology and Toxicology, University of Nairobi, Nairobi, Kenya; Fort Collins, UNITED STATES

## Abstract

**Background:**

Zoonoses account for most of the emerging and re-emerging infections in Kenya and in other low to medium-income countries across the world. The human-livestock-wildlife interface provides a nexus where transmission and spread of these zoonotic diseases could occur among communities farming in these areas. We sought to identify perceptions of the community living near the Lake Nakuru National Park in Kenya.

**Methods:**

We used participatory epidemiology techniques (PE) involving Focus Group Discussion (FGD) among community members and Key Informant Interviews (KII) with the health, veterinary, and administration officers in July 2020. We used listing, pairwise matching, and proportional piling techniques during the FGDs in the randomly selected villages in the study area from a list of villages provided by the area government officers. Kruskal–Wallis test was used to compare the median scores between the zoonotic diseases, source of information, and response to disease occurrence. Medians with a z-score greater than 1.96 at 95% Confidence Level were considered to be significant. Content analysis was used to rank qualitative variables.

**Results:**

We conducted seven FGDs and four KIIs. A total of 89 participants took part in the FGDs with their ages ranging from 26 to 85 years. Common zoonotic diseases identified by participants included anthrax, rabies, and brucellosis. Anthrax was considered to have the greatest impact by the participants (median = 4, z>1.96), while 4/7 (57%) of the FGDs identified consumption of uninspected meat as a way that people can get infected with zoonotic diseases. Community Health Volunteers (Median = 28, z = 2.13) and the government veterinary officer (median = 7, z = 1.8) were the preferred sources of information during disease outbreaks.

**Conclusion:**

The participants knew the zoonotic diseases common in the area and how the diseases can be acquired. We recommend increased involvement of the community in epidemio-surveillance of zoonotic diseases at the human-wildlife-livestock interface.

## Author summary

Diseases that can be transmitted between human beings and other domestic and/or wild animals are a growing concern to communities and other public health stakeholders. These diseases (zoonoses) can be found in the animal or human populations but interaction between humans and animals, especially at the Human-Livestock-Wildlife interfaces leads to transmission of these diseases between ecosystems. The socio-economic and health burden arising from the zoonoses are mostly experienced among poor communities with limited resources for early detection, control and management of the zoonoses in humans and animal. Implementation of control strategies for zoonoses have largely been left to the governments, though community members can play a role too. This perception is largely due to poor direct participation or inclusion of the communities in disease surveillance and control. This study identified community perceptions on zoonotic diseases and performed a qualitative risk assessment for people exposed to zoonotic pathogens. Our findings could be useful in designing integrated community-based strategies for surveillance and control of zoonotic disease at the human-livestock-wildlife interfaces.

## Introduction

Zoonoses are diseases and infections that are naturally transmitted between vertebrate animals and humans [1]. They account for most of the emerging and re-emerging infections in Kenya and other low and medium-income countries (LMICs) across the world. Most of the zoonotic diseases are considered neglected globally. The human-livestock-wildlife interface provides a nexus where transmission and spread of these zoonotic diseases could occur, especially in areas where agricultural intensification and encroachment into the wildlife territories occur [2]. Some of the listed priority zoonoses in Kenya include rabies, brucellosis, anthrax, and Rift Valley Fever [3]. These diseases cause morbidity, mortality, and adverse effects on the socio-economy and livelihoods, especially to those who are dependent on the livestock sector. In Kenya, these diseases have been reported in almost all parts of the country, with the rift valley and central Kenya regions reporting most of the cases [3,4]. Communities that practice crop and livestock farming close to the wildlife areas are particularly prone to contracting zoonotic diseases, some of which may originate from wildlife [2,5].

Nakuru West Sub County borders the Lake Nakuru National Park (LNNP), putting the community living at the interface with the park at risk of contracting zoonotic diseases. The area has had a recurrence of zoonotic diseases, including but not limited to anthrax over the recent past, with the latest occurring among the wildlife in the Lake Nakuru National Park, where it was reported that 10 buffaloes died of the disease [6,7]. Other zoonotic diseases that have been reported in the area include rabies and brucellosis.

Most studies conducted in the area have focused on the quantitative aspects of the disease and the risk factors associated with outbreaks of zoonoses. Other studies have provided information on the implementation of control strategies, though community members have perceived these strategies to belong to the government, whereas the communities could play a bigger role in the prevention and control of the diseases. This perception is largely due to poor direct participation or inclusion of the communities in disease surveillance and control. This participatory study sought to identify community perceptions on the diseases and perform a qualitative risk assessment for people exposed to zoonotic pathogens. Findings from the study will be used to propose ways of integrating community perception in designing the prevention of exposure to zoonotic disease strategies and contribute to surveillance of the diseases. This study assessed the community perception and risk of exposure to common zoonotic diseases in Nakuru West Sub County, Nakuru County.

## Materials and methods

### Ethics statement

This was a programmatic activity by the Kenya Ministry of Health and Kenya Directorate of Veterinary Services as part enhancement of surveillance for zoonotic diseases. We obtained verbal formal consent to eligible study participants to take both written and audio-recorded notes and the confidentiality of the information from the participants was maintained through the use of codes and password-protected computers and databases. Before starting each FGD, the participants were allocated numbers that were used to identify each one. Participants were informed of their right to withdraw at any stage of the interview in case they felt uncomfortable with the interview.

### Description of the study area

**Fig 1:**
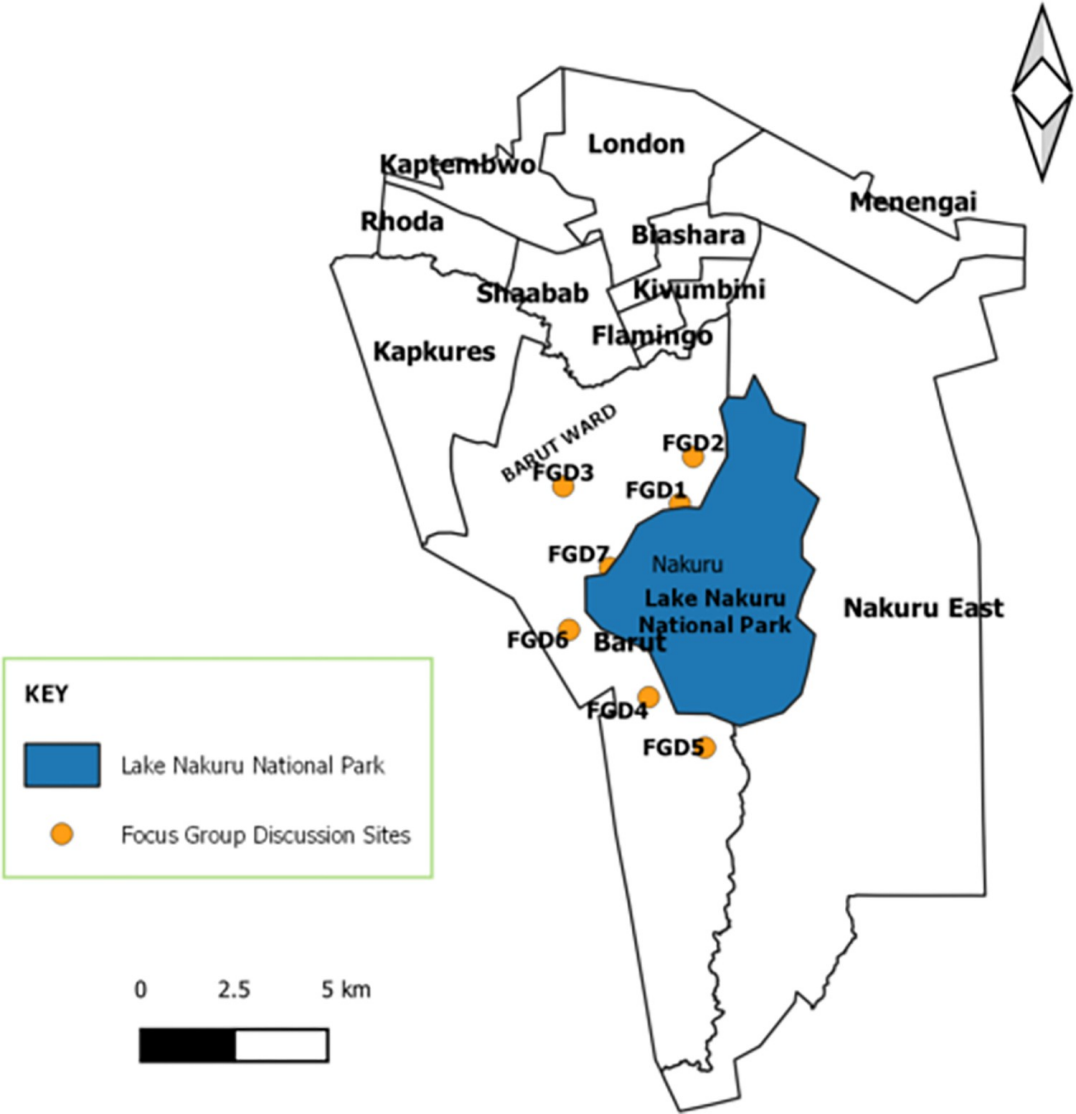
Study Area indicating Focus Group Discussion Sites bordering the Lake Nakuru National Park, Nakuru, Kenya, 2020. Figure generated by the authors using data from this study, an Open-Source Software, QGIS (qgis.org/en/site/), publicly available shapefile (https://openafrica.net/sv/dataset/kenya-counties-shapefile), licence available at; https://creativecommons.org/licenses/by/4.0/.

The study was conducted in Barut and Kaptembwa wards of Nakuru West Sub County in Nakuru County (Fig 1). The two wards border Lake Nakuru National Park and have a combined area of 200.6 km^2^ with a human population of 87,141 [8]. Most parts of Kaptembwa ward are peri-urban while most of the Barut ward is rural. Other wards in the Sub County include, London, Kapkures, Rhoda, and Shabab, covering a total of 251 km^2^ The area has predictable weather patterns with temperatures ranging between 10°C during the cold months (July and August) and 20°C during the hot months (January to March) with about 700–1200 mm of rainfall annually. The area is predominantly inhabited by communities practicing different forms of agricultural activities involving crop and livestock farming. The main livestock reared include cattle, poultry, sheep, and goats. The production system in the sampled population is semi-intensive [9] with farmers practicing semi-grazing and occasionally get pasture/fodder from areas adjacent to the Nakuru National Park. The dairy industry is the leading livestock enterprise with milk sold through the local market as well as cooperatives. Other smaller enterprises in the livestock sector include poultry, beef, and the emerging livestock species such as quails, pigeon, ostrich, honey, and hides and skin [10].

### Study approach

We used participatory epidemiology techniques (PE) involving Five (5) Focus Group Discussion (FGD) comprising 12–15 participants per FGD and four (4) Key Informant Interviews (KIIs) in July 2020. Participants in the FGDs were community members, while those in KIIs were human healthcare workers, veterinary personnel and the local administration officers. We randomly selected seven villages that border the Lake Nakuru National Park (LNNP) in the study area from a list of 15 villages provided by the local administration officers. The villages selected were Subuku, Mwariki, Sossiot, Kimolwet, Kigonor, Flamingo, and Kelewet. Purposive selection of participants was done with the help of the area administration, public health, and veterinary officers to identify people who could give information on zoonotic diseases and predisposing practices to zoonotic diseases in the study area. A participant had to be an adult residing in the selected villages and either come from a household that kept livestock or trade in animals or products of animal origin along the livestock value chains. Each FGD had both men and women in almost equal proportions. Each FGD was conducted for about 2 hours in Kiswahili at centrally located common grounds within each selected village.

### Data collection

Data collection was done by the use of open-ended questions in a focused group discussion. We adopted the guidelines from the Food and Agricultural Organization (FAO) manual for conducting participatory epidemiology (PE) activities [11]. We used PE techniques of semi-structured interviews, listing, pairwise ranking, and proportional piling. Probing techniques were used to obtain quality data and ensure consistency of information from the different FGDs. The information from each FGD was recorded using handwritten notes, flip charts, manila papers, felt pens, and voice recorders. Data collected included diseases frequently encountered in the community, diseases with high impact based on effects on livestock production parameters, cost of treatment, morbidity and mortality. Other variables included community practices that expose the people to infection with zoonotic diseases, reporting of occurrence of livestock diseases, sources of information on outbreaks of livestock diseases, reduction of risk of people getting infected by zoonotic diseases and community response to the occurrence of livestock diseases. Global Positioning System (GPS) gadgets were used to obtain coordinates for each FGD site for the generation of locational maps of the FGD sites using Quantum GIS software (QGIS Development Team)

### Common zoonotic diseases and predisposing practices

To assess the level of importance accorded to specific zoonotic diseases, the participants were asked to list zoonotic diseases they considered to be common in the area. The participants were allowed to use the local names of the diseases and where there was no common local name, the key signs, and symptoms of the diseases were used. The local veterinary and human health personnel, who understood the local language, ensured that the local names given by the participants were consistent with the official names of the diseases. The participants then performed pairwise ranking where participants were asked to discuss and compare the effects of every two diseases at a time based on human and livestock mortality, morbidity, and socio-economic losses. The names of diseases were written vertically (y-axis) and horizontally (x-axis) in columns and rows on a flip chart. For each pair of diseases (x,y) participants were asked which disease was more important as the investigation team probed why they felt it was important than the other. For each pair of diseases compared, A mark (1) was given to the disease considered more important, and no mark (zero) given to the disease considered less important. The scores were then tallied for each disease and ranking done based on the scores. The participants then listed community practices that expose them to infection with zoonotic diseases. Content analysis and proportions were determined to identify the common practices across the groups.

### Source of Information during disease outbreak

To identify the preferred source of information during zoonotic disease outbreaks, the participants were asked to list the various sources of information that they have. Proportional piling technique was then used to identify the most preferred source of information by listing the sources in a flip chart and each group given 100 beans to distribute across information sources based on their relative importance with the most preferred information source being allocated the highest number of beans. The participants then counted the number of beans allocated to each information source to obtain the scores for each information source. Probing was used to discuss the scores and to obtain reasons that informed the allocations. Each group was then asked to list how they would respond in the event a zoonotic disease occurred in their farm or neighborhood. Proportional piling and scoring were then conducted for the listed responses as explained above.

### Key Informant Interviews

Interviews were conducted using open-ended questions to the area veterinary officer including the officer in charge of the regional veterinary investigation laboratory, the area public health officer, and the area administration officers. Information obtained from the KIIs included political support in disease control and prevention, main sources of livelihoods for the community, how the communities relate with animals/livestock and their products. In addition, the key informants responded to questions on technologies available for use in disease reporting, vaccines and medicines availability for priority diseases, the legal framework for use in disease control and prevention.

### Data management and analysis

Scoring and ranking data obtained from the focus group discussions were entered in a workbook then exported for analysis using descriptive and non-parametric statistical methods. The analysis involved computing proportions, medians, and z-scores. Kruskal–Wallis test was used to compare the median scores between the zoonotic diseases, source of information, and response to disease occurrence. Medians with z-score greater than 1.96 at 95% Confidence Level were considered to be significant. Content analysis was used to rank the practices and reporting of disease occurrence at the farm level.

## Results

### Knowledge of livestock farmers on zoonotic diseases and their response measures

The participants in the focus group discussions identified some diseases that are zoonotic and can be transmitted between their livestock and members of their households. The diseases which were commonly reported across the group discussions included but not limited to anthrax, Swine Respiratory Diseases, pneumonia, Multiple abscesses, rabies, and brucellosis (Table 1). Of the listed zoonotic diseases, anthrax (Z = 4.23) and Swine Respiratory Diseases (Z = 2.30) were ranked higher than the other diseases in terms of community knowledge on the diseases. Anthrax is a disease which, according to them, was frequently reported in the adjoining regions to the Nakuru national park where the disease had recently killed wildlife and sometimes the livestock. Cysticercosis which could be due to the people’s interaction with pigs within the informal settlement, was also reported in the region by focus group participants, although it ranked low in importance.

**Table 1:** Zoonotic diseases identified by participants in the focus group discussions, Nakuru, Kenya, 2020.

Diseases identified by participants	Median rank	Average rank	Z score
Anthrax	4.00	73.10	4.23
Swine Respiratory Diseases	3.00	57.60	2.30
Pneumonia	2.00	54.60	1.94
Multiple abscesses	1.00	44.10	0.64
Myiasis	0.00	34.60	-0.55
Rabies	0.00	31.60	-0.92
Avian Flu	0.00	28.80	-1.27
Worms	0.00	28.80	-1.27
Brucellosis	0.00	27.80	-1.39
Cysticercosis	0.00	24.00	-1.86
Tetanus	0.00	24.00	-1.86

Farmers who participated in the FGDs indicated that whenever disease occurred in their herds, they responded by isolating the animal from the rest of the herd, observing the progression of the disease, and treatment with local herbs (Table 2). After these preferable measures fail, they would only report to the veterinary officers or take other actions. Of all the measures practiced by these farmers, isolation of the sick animals (Z = 2.46) and observation of these sick animals (Z = 1.99) were mostly practiced in farms. There were several predisposing factors for exposure to zoonotic diseases by the farming communities in Nakuru (Table 3).

**Table 2:** How Participants would respond to the Occurrence of Diseases, Nakuru, 2020.

Response to disease occurrence	Median	Average rank	z- score
Isolating sick livestock	3.00	53.4	2.46
Observing the sick livestock	2.00	50.0	1.99
Treatment using local herbs	2.00	43.7	1.13
Reporting to veterinary officers	1.00	41.8	0.86
Calling private animal health service provider	0.00	32.1	-0.47
Calling government animal health service provider	0.00	31.6	-0.54
Reporting to Agro-veterinary shop owner	0.00	28.4	-0.97
Taking sample to Veterinary Laboratory	0.00	26.8	-1.19
Slaughtering for home consumption	0.00	25.7	-1.34
Consulting Experienced farmer	0.00	21.5	-1.92

H = 22.99 (adjusted for ties) with 9 d.f, Probability > 22.99 = 0.0062 n; number of groups, z; Z score at 95% Confidence Level

**Table 3:** Farm-level practices that expose people to infection as identified by focus group participants, Nakuru, Kenya, 2020.

Predisposing factors for exposure to zoonoses at farm level	Flamingo	Kelewet	Kigonor	Kimolwet	Mwariki	Sossiot	Subuku
Poor hygiene leading to worm infestation	√	√	√	√			√
Drinking of un-boiled milk	√	√				√	√
Not deworming livestock	√	√		√	√		
Eating uninspected and infected meat	√	√	√		√		
Consumption of improperly cooked meat	√	√	√		√		
Poor human waste disposal	√	√	√				
Not vaccinating livestock	√	√		√			
Ignorance or lack of concern			√	√		√	
Handling infected carcass					√	√	√
Lack of vaccination for pets					√	√	√
Contact with infected products	√	√					
Grazing of animals in infected areas	√	√					
Improper disposal of carcass					√		√
Close contact with livestock			√				
Giving carcasses to dogs			√				
Consumption of game meat			√				
Crowding/common grazing				√			
Poor fencing/poor containment				√			
Delay in seeking veterinary services						√	
Contaminated/infected feed						√	

### Predisposing factors for zoonotic diseases and disease outbreak information sharing measures

Most of the groups identified poor hygiene practices in farms, resulting in worm infestation as the main factor of exposure to zoonoses in the region. The other factors identified by farmers, and were often practiced in at least three groups, included drinking of un-boiled milk; not deworming livestock; eating uninspected (pre and post-slaughter inspection) and infected meat by households. Consumption of improperly cooked meat, poor human waste disposal due to lack of latrines, not vaccinating their livestock against diseases, handling infected carcasses and lack of vaccination of pets were also identified as predisposing factors. The other risk practices included contact with infected carcasses or products, grazing animals in infected areas, improper disposal of a carcass, giving carcasses to dogs, and consuming game meat. Indeed, close contact with infected carcasses and their consumption increases the risk of exposure to the farming communities.

For the most part, disease reporting by the communities was through the agro-veterinary pharmacist outlets, which are businesses that sell agricultural inputs to farmers, local governmental officials based at the grassroots, public veterinary officers, and ministry of health officials (Table 4). The other modes of reporting outbreaks were through village elders, community health volunteers, public health officers and opinion leaders who could be experienced farmers who live within the region.

**Table 4:** People to whom the focus group participant would report disease occurrence in their farms to in Nakuru, Kenya, 2020.

Report disease occurrence to	Flamingo	Kelelwet	Kigonor	Kimolwet	Mwariki	Sossiot	Subuku
Agro-veterinary pharma outlets	√	√	√	√	√	√	√
Local government administration	√	√	√		√	√	√
Public veterinary officers	√	√	√	√		√	
Ministry of Health Officers (MOH)	√	√		√	√	√	
Private veterinary practitioners					√	√	√
Private Medical practitioners	√	√					
Village elders (Nyumba Kumi leaders)			√		√		
Community Health Volunteer (CHV)			√				
Public Health Officer (PHO)			√				
Chemist shop						√	
Report to experienced farmer							√
Private medical practitioner							√

The most frequently used methods for sharing information on disease occurrence to the livestock farmers were through community health workers, veterinary officers, local chiefs, and village elders (Table 5). Other technology approaches to reach the farmers included mass media (radio and television advertisements), posters, and social media platforms. Although the farmers did not rank the community health workers as the people they would readily share disease outbreaks with from their farms, this group of experts was often used to pass information on disease occurrence within the area by the government.

**Table 5:** Source of Information during Disease Outbreaks, Nakuru, Kenya, 2020.

Sources of information during disease outbreaks	Median	Ave rank	z- score
Community health volunteers	28.00	61.30	2.13
Veterinary officer	7.00	58.40	1.80
Local administration (chief)	6.00	49.50	0.79
Public address	5.00	45.60	0.35
Village elders	3.00	45.20	0.31
Farm products consumers	4.00	43.90	0.15
Churches	0.00	41.50	-0.11
Mass media (radio, television)	0.00	39.40	-0.36
Social gatherings (barazas)	0.00	36.70	-0.66
Fellow farmers	0.00	33.90	-0.98
Posters	0.00	28.10	-1.63
Social Media (WhatsApp, twitter)	0.00	26.60	-1.80

H = 17.02 (adjusted for ties) with 11 d.f Probability > 17.02 = 0.1072, n = number of groups, Z = Z score at 95% confidence Level

### Surveillance of zoonotic diseases

A key informant from the veterinary office stated that ’*the area is vast and is currently being served by one officer who cannot be accessed by so many residents at the same time*’. The area administrator said that ’*our people love meat and go to greater lengths to get meat even if it is from dead animals including wild animals like the buffaloes from the national park*.’ One of the key informants from the regional veterinary investigation laboratory (RVIL) stated that the causative agent for anthrax, *Bacillus anthracis*, had been detected in some of the samples from the study area in the preceding years. The officer from the RVIL indicated that serological disease search for Rift Valley fever had shown the presence of the disease in the study area. However, none of the FGDs included RVF or its symptoms during the FGD activities as a prevalent disease in the area. The veterinary officer indicated they use the Kenya Animal Bio-Surveillance System (KABS) whose data originate from the animal health service providers who had been trained on the use of the system.

The public health officer (PHO) in the study area stated that there is legislation in the public health act chapter 242 of the laws of Kenya [12] that could be used in the prevention of consumption of uninspected meat. The PHO indicated that the health department uses the online-based District Health Information System (DHIS) for disease reporting with the most reports originating from the community through the CHVs. Data for Nakuru West Sub County (District) from the DHIS from 2015 through 2019 indicated that human brucellosis (1634 cases) was a common zoonotic disease while human anthrax (zero cases) and human RVF (zero cases) were not common in the area (Table 6).

**Table 6:** Zoonotic Diseases reported in the District Health Information System, Nakuru West Sub County, 2015–2019.

Year	Brucellosis	Dog Bites	Intestinal worms	Taenia spp.	Rift Valley Fever	anthrax	Snake Bites	Tetanus	Trypanosomiasis
**2015**	330	1	1015	9	0	0	1	0	0
**2016**	255	97	2630	2	0	0	48	0	0
**2017**	172	244	1213	151	0	0	55	6	0
**2018**	492	455	6396	15	0	0	14	2	2
**2019**	385	450	1884	17	0	0	11	0	0
TOTAL	1634	1247	13138	194	0	0	129	8	2

## Discussion

This participatory study revealed several zoonotic diseases in the study area and that the community knows how they can be transmitted from animals to humans. The participant identified anthrax and brucellosis as common in the area, an assertion supported by findings from other studies that reported the occurrence of anthrax in the area and the park [4,7]. It should be understood that the identification of the disease in this study should be taken with caution, as there may be misclassification because no clinical or laboratory test was used to back the claims. The practice of consuming meat from animals that may have died from zoonotic diseases in the study area could be contributed by the moderately high poverty index [13]. Most of the residents practice crop farming, with few of them keeping livestock. Because of this observation, animal-based protein may sometimes be scarce, thus prompting the consumption of meat from carcasses with doubtful causes of death [14].

Most of the participants preferred reporting animal disease occurrence to privately owned agro-veterinary pharmaceutical outlets; however, it was worth noting that several residents would also report to government officers like the veterinary officers and the area administration officers. To trigger prompt action geared towards the prevention of the spread of disease, residents must engage the national and the sub-national levels of government. Such engagement would also help enhance surveillance and early detection of emerging and re-emerging diseases, as was highlighted in an assessment done by the National Academy of Sciences [15]. It is also imperative for the animal health authorities to recognize society’s contributions, including the private sector, in animal disease reporting. There is a great synergy when the community is included in the surveillance and reporting of disease incidence to relevant authorities, a key in early detection, response, and mitigation of emergence and re-emergence of infections [16]. The agro-veterinary pharmaceutical outlets that the participants preferred due to ease of accessibility could also provide quality data on diseases in the area. Most of the outlets are operated by animal health service providers licensed by the Kenya government. They are expected to report occurrence of diseases to the relevant authorities as outlined in the regulations [17].

Information flow during disease outbreak situations is very key but can be chaotic during communication to the public on the signs and symptoms of diseases, particularly a novel emerging disease, mode of transmission and preventive and control measures. Our findings indicated that the participants’ most-preferred method of getting information during outbreaks is through the use of public address systems from the administration officers and the veterinary officers. In most instances, the government officers spread information through the public address system to reach a wider area. Information from government health providers are taken more seriously, especially during disease outbreaks, thus preferable use of this system would be ideal, especially in areas with low literacy levels, where residents may not read posters or have access to radio or television used in mass media as indicated in studies done in other regions of the world [18,19]. Pictorial rather than literal messaging may also be beneficial for this purpose [15].

Access to a health service provider during disease outbreaks is of great importance to the livestock owners since providing drugs and advice goes a long way in curbing the spread of diseases and reducing economic losses at individual farmer level or at the society level [20]. The participants indicated that their first point of call in the event of disease occurrence would be the nearest agro-veterinary pharmaceutical outlets since a professional/paraprofessional is available to advise and give drugs at affordable prices. The participants also stated that they would prefer calling the private veterinary service provider due to their accessibility at short notice compared to government veterinary service provider, who typically take a longer period to respond. The government veterinarians are few and the ones available cover a wide area in their jurisdiction. Zoonotic diseases are of high economic and public health impacts during outbreaks, as they are considered as highly contagious livestock diseases, hence mitigation measures should be instituted at the earliest opportunity [16]. It would be prudent to promptly offer these measures to stop the slaughter and consumption of sick animals as some residents who use traditional herbs to treat sick animals tend to consume such animals as a last resort if the treatment uses herbal medicine, or sometimes antimicrobials failed [21,22]. The false confidence that self-medication by the farmer is beneficial and that should it fail, the consumption of the carcass post-slaughter is alright may lead to transmission of zoonotic pathogens to humans through the food chain.

The residents in the study area had good access to food crops with limited access to food of animal origin. Hence, the resort to consuming sick or dead animals and edible wild animals that may stray into the human settlement, including the captured ones, was natural for the community. For instance, a participant stated:

’*if a buffalo stray into my compound and I kill it*, *that will be like a blessing to me and my people*, *since we will have rare opportunities to eat meat*’.

The area administrator confirmed that such scenarios had been seen before, leading to disastrous consequences to the families, for instance, cases of anthrax in humans. This scenario calls for enhanced surveillance and the implementation of a community-level robust reporting system that supports prompt reporting of any diseases with zoonotic potential, more so if the occurrence is detected at the wildlife-livestock-human interphase [23,24]. The integration and involvement of the community in the surveillance of diseases through establishment of community One Health Units, with structured leadership and roles, could be useful channel for prevention of the spill-over of pathogens from animals to humans [12,19,20].

## Conclusion and recommendation

The study area residents were aware of common zoonotic diseases in their locality and how animals can transmit or contract the conditions. We recommend the community’s involvement in the surveillance of zoonotic diseases at the human-wildlife-livestock interphase by integrating it into the existing surveillance systems. Such involvement may trigger a sense of responsibility and ownership among the community members and create a semblance of community policing that may enhance the surveillance of zoonotic diseases.
